# The Use of Bioadditives as Plasticizers in Recycled Polyethylene Materials

**DOI:** 10.3390/ma19030570

**Published:** 2026-02-02

**Authors:** Kalina Joanna Kaczmarek, Justyna Miedzianowska-Masłowska, Marcin Masłowski

**Affiliations:** Institute of Polymer and Dye Technology, Lodz University of Technology, Stefanowskiego 16, 90-537 Lodz, Poland; 243210@edu.p.lodz.pl (K.J.K.); marcin.maslowski@p.lodz.pl (M.M.)

**Keywords:** recycling, polyethylene composites, biochar, plasticizer, composites, natural substances

## Abstract

The growing emphasis on sustainable material design has intensified interest in bio-based additives as environmentally friendly alternatives to conventional synthetic modifiers. This study evaluates the effects of four natural compounds—cetyl alcohol, thymol, lanolin, and lecithin—on the thermal, rheological, mechanical, surface, and aging properties of regranulated low-density polyethylene (RLDPE). Post-consumer polyethylene waste was used as the polymer matrix, while biochar served as a sustainable reinforcing filler replacing carbon black. Differential scanning calorimetry, melt flow index measurements, rheological behavior, surface energy analysis, mechanical testing and thermo-oxidative aging assessments were conducted to assess structure–property relationships. Biochar increased stiffness, hardness, and impact resistance but reduced ductility and melt flow due to restricted chain mobility. The addition of natural compounds partially compensated for these effects by improving melt flow, modifying crystallization behavior, and enhancing resistance to thermo-oxidative degradation without severely diminishing mechanical performance. Cetyl alcohol promoted the highest crystallinity and flexural properties, lanolin exhibited the strongest plasticizing effect and improved post-aging ductility, while lecithin and thymol produced intermediate changes, with lecithin significantly increasing surface energy. These results indicate that selected natural additives can act as effective ecological plasticizers or processing aids in biochar-filled recycled polyethylene composites.

## 1. Introduction

Thermoplastics constitute one of the most important classes of materials currently employed [1]. The advantageous properties of these materials include the ability to be repeatedly softened under the influence of temperature and subsequently remolded without significant loss of performance, as well as their favorable strength-to-weight ratio, high chemical resistance and relatively low production costs [2,3,4]. Owing to these characteristics, thermoplastics are widely applied in the manufacture of automotive components, electronic devices, and medical products [5]. Thermoplastics are also employed in the packaging industry, where they rank among the most commonly used materials due to their low production cost [6,7]. However, the combination of large-scale production and the limited degradability of thermoplastics in the natural environment has led to a continuous accumulation of polymers [8,9], which represents a major ecological and economic challenge [10,11,12]. In response to increasing sustainability requirements and the introduction of stricter legal regulations, research efforts have recently intensified towards the development of alternative materials, including modified recyclates and biocomposites [13,14]. The primary objective of these studies is to preserve the desirable functional properties of thermoplastic-based materials, while simultaneously mitigating their adverse environmental impact [15].

A promising direction in material design involves the use of additives characterized by lower toxicity, greater reliance on renewable raw materials, and a reduced environmental footprint [16,17]. Conventional, non-renewable additives, such as carbon black, are being progressively substituted with alternative fillers of natural origin. An example of such a substance is biochar, a material obtained through the pyrolysis of biomass [18]. This material exhibits a high specific surface area and low toxicity [19]. The utilization of biochar as a filler in polymer composites has been demonstrated to reduce the consumption of fossil resources, while simultaneously imparting additional functional properties to the materials, thereby lowering their environmental impact [20,21].

In the field of polymer science, traditional plasticizers play a significant role in improving the flexibility and processability of polymers [22]. This is particularly relevant in the context of recycled polyethylene composites modified with biochar. The recycling process, which involves polymer chain scission, and the introduction of biochar particles, lead to increased stiffness of the material [23]. This increase in stiffness deteriorates the composite’s processing properties. Consequently, the utilization of plasticizers becomes essential for restoring the desired flexibility and optimizing overall performance. Traditional plasticizers are based on petrochemical compounds, such as phthalates or adipates, which carry the risk of migration into the environment, exhibit low biodegradability, and pose potential toxicological concerns [24]. In response to these challenges, there is growing interest in natural substances obtained from vegetable oils, sugars and fatty acids, among others [25,26]. These bio-based plasticizers are reported to exhibit a more favorable ecological profile and a reduced impact on human health [25].

The purpose of this study is to determine the impact of selected additives on the mechanical and functional properties of recycled polyethylene film modified with biochar. Despite the beneficial influence of biochar on the thermal stability, ultraviolet (UV) resistance, and barrier properties of the polymer matrix, its incorporation simultaneously induces a significant increase in stiffness [18,27,28]. Such stiffening, when further intensified by the effects of the recycling process, contributes to a pronounced deterioration of the material’s flexibility. To counteract this limitation, natural plasticizers were introduced into the system, namely thymol, lanolin, lecithin and cetyl alcohol, with the objective of enhancing the processing properties of the material. The choice of these particular plasticizers was guided not only by their expected effectiveness in improving the mechanical performance of the composite but also by their renewable origin, which ensures consistency with the principles of environmentally sustainable material design.

The results obtained provide valuable insights that may substantially support the development of more environmentally friendly thermoplastics, particularly in the context of polyethylene waste management and its modification using natural fillers. Demonstrating the reinforcing potential of biochar, alongside elucidating the influence of natural plasticizers such as thymol, lanolin, lecithin, and cetyl alcohol on the mechanical and functional characteristics of the composite, enables the formulation of materials containing a reduced proportion of petrochemical-based additives and exhibiting a lower environmental burden. This approach is consistent with the principles of a circular economy, which prioritizes the reuse of plastic waste and biomass resources. Moreover, these findings advance the current state of knowledge regarding the application of natural plasticizers in the materials science domain, supporting their rational selection for tailoring specific polymer properties without compromising mechanical integrity or long-term durability. It is hypothesized that, in the long term, such strategies may contribute to reducing pollutant emissions, decreasing reliance on fossil-derived raw materials and promoting of environmentally oriented innovations within the polymer industry.

This study introduces a novel approach by combining biochar reinforcement with natural additives—cetyl alcohol, thymol, lanolin, and lecithin—in recycled low-density polyethylene, systematically assessing their effects on thermal, rheological, mechanical, and surface properties. Recent research has increasingly explored the use of biochar as a sustainable reinforcement filler in thermoplastic and elastomeric matrices, demonstrating improvements in mechanical and thermal performance compared to unfilled or conventionally filled polymers, and highlighting its potential to replace petroleum-derived carbon black in composite formulations [29]. Studies have shown that biochar incorporation can enhance tensile strength, modulus, and thermal stability in a range of polymer systems, supporting its role as an eco-friendly alternative filler within circular materials strategies. Similarly, bio-based additives and natural compounds are gaining traction as functional modifiers due to their ability to influence processing behavior and end-use properties while reducing reliance on synthetic stabilizers; reviews on sustainable plasticizers and natural antioxidants in polymer composites underline the trend toward incorporating renewable, low-toxicity additives to tailor material performance [30]. The results highlight how bio-based additives can counteract stiffness, enhance processability, and tailor material performance, offering a sustainable strategy that supports both high-performance polymer design and circular economy principles through the valorization of post-consumer polyethylene waste.

## 2. Materials and Methods

### 2.1. Preparation of the Biofiller and Composition of the Mixtures

Biochar (BC) used in the composites was sourced from bamboo charcoal provided by Zuzii (Sękocin Stary, Poland). The material was ground in a SPEX SamplePrep 8000D planetary mill (Metuchen, NJ, USA), to obtain a fine fraction.

The polyethylene film with a molecular weight of 10^5^–10^6^ g/mol, was manually cut and fed into the extrusion hopper. Processing was conducted using a laboratory ZAMAK Mercator single-screw extruder (Skawina, Poland). This was configured with three heating zones, set at 195 °C, 190 °C and 180 °C. The initial zone functioned to soften the feedstock, the second promoted homogenization and degassing, and the third stabilized pressure prior to strand discharge. The extruded strand was then granulated using a Brabender strand granulator (Duisburg, Germany). The compositions of the polyethylene mixtures used for further tests are presented in Table 1. All of the mixtures contained 100 parts by weight (pbw) of RLDPE.

The compound preparation process was divided into two stages. In the first stage, polyethylene granulate was mixed with the biofiller and mechanically pre-mixed plasticizers, namely lecithin (LE), lanolin (LA), cetyl alcohol (CA), and thymol (TYM). The formulations of all composites are expressed in pbw (parts by weight) of PE. In practice, this means that each sample was prepared with 100 g of polyethylene, and the respective amounts of other ingredients were calculated accordingly, as shown in Table 1. Higher contents (10 pbw) were used for lecithin and lanolin due to their high molecular weight and lower mobility, while lower amounts (5 pbw) of cetyl alcohol and thymol were selected to avoid excessive softening or potential migration, while still allowing their functional effects on mechanical and aging behavior to be evaluated. In the subsequent stage, the mixtures were once again placed in the extruder and granulated under the same temperature conditions as the first stage.

### 2.2. Injection Molding of the Composites

Samples for mechanical testing were produced using the injection molding method on a Battenfeld PLUS 350 machine (Kottingbrunn, Austria). Initially, the reference material was processed, followed by the composites. Each series was subjected to analysis separately, and the cylinder was cleaned between cycles to avoid contamination.

**Figure 1:**
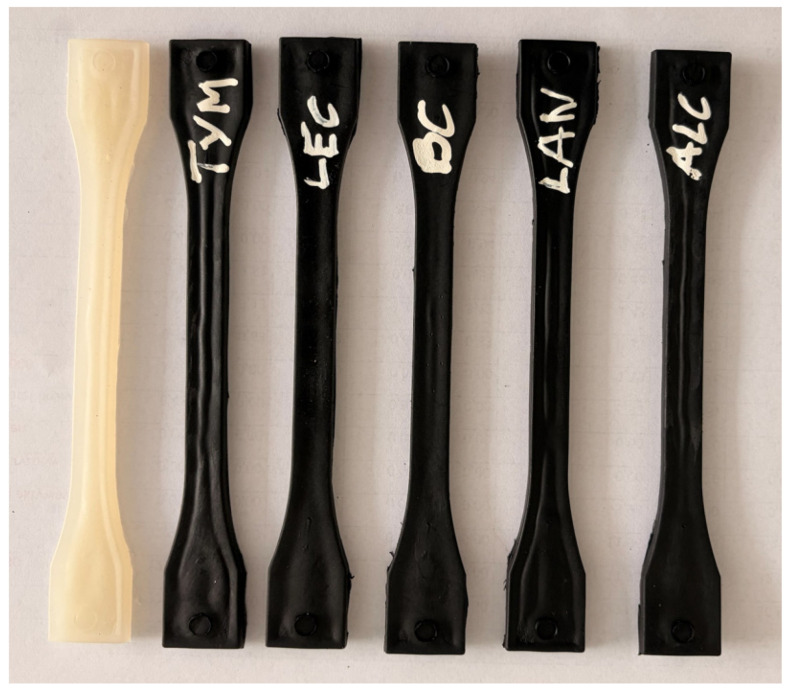
Photographs of neat polyethylene (PE) and polyethylene-based composite samples containing biochar (BC) and selected natural additives. From left to right: PE, PE with biochar and thymol, PE with biochar and lecithin, PE with biochar, PE with biochar and lanoline, and PE with biochar and cetyl alcohol.

The injection itself was carried out in a five-stage cycle. The sequence began with the closure of the mold, followed by the retraction of the plasticizing unit to fill the material, and then the unit was pushed forward again. This was followed by the injection of the polymer with a short compression stage. Thereafter, the mold was opened, and the finished part was removed. The samples were produced under a two-stage temperature profile of 210 °C in the cylinder and 220 °C at the nozzle. This resulted in the production of molded samples in the shape of blades and beams, which were then subjected to mechanical testing. The samples obtained are shown in Figure 1.

### 2.3. Determination of the Melt Flow Index (MFI)

The melt flow rate (MFR) and melt volume rate (MVR) were determined in accordance with PN-EN ISO 1133-1 [31] using a Melt-Flow Plus tester (Karg Industrietechnik, Krailling, Germany). The experimental procedure was conducted at a temperature of 190 °C, with a load of 2.16 kg, following a preheating period of 240 s. A quantity of 4 g of granules was placed in the cylinder. The apparatus recorded the extrusion time and determined the MFR (g/10 min) and MVR (cm^3^/10 min) values.

### 2.4. Viscosity of the Composites

Rheological measurements were performed using an ARES-G2 rotational rheometer (TA Instruments, New Castle, DE, USA). Test samples were prepared by subjecting weighed portions of granules to compression in a heated hydraulic press, and then cutting them into disks with a diameter of 25 mm and a thickness of approximately 2 mm.

To study the dependence of viscosity on temperature, the prepared disks were placed in the rheometer. At a constant shear rate, the sample was heated from 100 °C to 250 °C, with a linear temperature increase of 5 °C/min. The apparatus was cleaned and then a fresh sample was placed in the measuring system to assess viscosity as a function of shear rate. At a constant temperature of 200 °C, the rheometer gradually increased the rotation speed.

### 2.5. Impact Strength

The impact resistance of the composites was determined by conducting a series of tests in accordance with the Charpy impact method, as specified in the PN-EN ISO 179-1 [32] standard. The measurements were performed using a Cometech QC-639P pendulum impact tester (New Taipei City, Taiwan). Prior to the experiment, the thickness of each beam was measured at multiple points using a caliper. The samples were then positioned on the supports of the device and subjected to impact with a Charpy hammer. Three measurements were taken for each material variant, and the result was expressed as the recorded Charpy impact strength (Eₛₐ) [kJ/m^2^].

### 2.6. Hardness

The test was conducted using a Zwick 3105 Shore durometer (Ulm, Germany). Standard injection-molded bar specimens from each formulation were placed in the measuring device. Five measurements were obtained for each sample by applying a needle with a rounded indenter tip to the test piece. The final result was expressed as the mean value of the obtained measurements.

### 2.7. Three-Point Bending

The three-point bending tests were conducted in accordance with the standard PN-EN ISO 178 [33], using a ZwickRoell RetroLine universal testing machine (Ulm, Germany). Following the application of a preload force of 0.1 N to the specimens, the crosshead was moved at a rate of 10 mm/min to ascertain the flexural modulus (Ef). Thereafter, the crosshead was accelerated to 50 mm/min to determine the maximum flexural stress (σfM).

### 2.8. Mechanical Properties of Composites Under Static Conditions

The test was conducted using a ZwickRoell 1435 universal tester (Ulm, Germany) in accordance with PN-EN ISO 527-1 [34]. The thickness of each specimen was measured individually prior to testing and entered into the ZwickRoell software. The following testing parameters were established:

- T_fmax_—true stress at maximum load
- E_fmax_—true strain at maximum load

### 2.9. Differential Scanning Calorimetry (DSC) Analysis

DSC measurements were performed using a Mettler Toledo DSC1 differential scanning calorimeter (Mettler-Toledo International Inc., Greifensee, Switzerland). The granules was placed in aluminum calorimetric pans specifically designed for this purpose, together with a reference pan, and introduced into the calorimeter chamber. The samples were initially exposed to a heating cycle from −150 °C to 200 °C at a constant rate of 10 °C/min, aimed at erasing their previous thermal history. The sample was then cooled to a temperature of −150 °C to observe the crystallization process. It was once again heated to 200 °C to determine the glass transition temperature and equilibrium melting enthalpy. Nitrogen gas was used as a protective atmosphere throughout the experiment to prevent oxidation.

The crystalline phase content (X_c_) of the samples was determined based on DSC measurements. The calculations were performed using the following equation:

$$X_{c}=(\frac{\Delta H}{\left(1-w_{f}\right)\Delta H_{0}})\times 100\%$$

where:

ΔH—the measured melting enthalpy of the sample (J/g), obtained from the area of the melting peak recorded during the DSC analysis,

ΔH_0_—the melting enthalpy of 100% crystalline polyethylene, assumed to be 293 J/g according to literature data [35],

w_f_—the weight fraction of filler.

### 2.10. Fourier-Transform Infrared Spectroscopy (FTIR) Analysis

Fourier-transform infrared spectroscopy (FTIR) was used to qualitatively analyze the chemical structure of the RLDPE-based composites and to assess possible interactions between the polymer matrix, biochar, and the incorporated natural additives (cetyl alcohol, thymol, lanolin, and lecithin). The FTIR spectra were recorded in the range of 4000–400 cm^−1^ with a resolution of 8 cm^−1^, averaging 64 scans per measurement. Measurements were carried out using a Thermo Scientific Nicolet 6700 spectrophotometer (Thermo Fisher Scientific, Waltham, MA, USA) equipped with a Smart Orbit ATR accessory.

### 2.11. Surface Energy Measurement

The surface energy measurements were performed using the sessile drop method with an optical goniomete (OCA 15EC, DataPhysics, Filderstadt, Germany). The surface energy was determined from the recorded images immediately after depositing 10 µL droplets of distilled water and diiodomethane on the surface of the composite.

### 2.12. Thermo-Oxidative Aging

The samples were placed in a thermo-oxidation chamber and aged at 70 °C for a period of 14 days in order to induce accelerated thermo-oxidative degradation. After completion of the aging procedure, the samples were conditioned to ambient laboratory conditions and subsequently subjected to mechanical testing. The obtained results were used to evaluate the influence of thermo-oxidative aging on the mechanical performance of the investigated material.

The aged samples were subsequently subjected again to functional characterization tests in order to evaluate the extent of degradation induced by the thermo-oxidative aging processes. The aging factor (K) was calculated based on the results of the mechanical strength tests, according to the following equation:

$$K=\frac{{Tf}_{after}\times {Ef}_{after}}{{Tf}_{before}\times {Ef}_{before}}$$

where:

Tf_after_—true stress at maximum load [MPa] after aging,

Ef_after_—true strain at maximum load [%] after aging,

Tf_before_—true stress at maximum load [MPa] before aging,

Ef_before_—true strain at maximum load [%] before aging.

### 2.13. Statistical Analysis and ANOVA

All measurements of melt flow index (MFI) and mechanical properties—including impact strength, hardness, tensile strength, and three-point bending—were performed in replicates. The reported values represent the mean of the measurements and are accompanied by standard deviations to indicate the variability and reliability of the data.

To assess the significance of observed differences between composite formulations, a one-way analysis of variance (ANOVA) was conducted. The statistical analysis confirmed that variations in carbon filler type and additive content led to statistically significant differences in specific properties (*p* < 0.05).

## 3. Results

### 3.1. Melt Flow Index

The rheological behavior of the composites was assessed by measuring the melt flow index (MFI), expressed as the melt volume rate (MVR) and melt mass rate (MFR). This parameter is critical for determining the processability of polymers, as it indicates the ease of flow in the molten state. The results of the measurements are presented in Table 2.

The incorporation of biochar into the polymer matrix reduced the MFI from 0.732 cm^3^/10 min (reference RLDPE) to 0.556 cm^3^/10 min, indicating an increase in melt viscosity. This result suggests that the rigid filler particles restrict the mobility of polymer chains, producing a more viscous and structurally stiffer melt. Similar reductions in MFI and corresponding increases in melt viscosity upon biochar addition have been reported in previous studies, where the effect was attributed to restricted polymer chain mobility and enhanced filler–matrix interactions [36].

Upon the incorporation of natural additives, the MFI values increase again, indicating a partial recovery of polymer chain mobility. This effect can be attributed to the lubricating or plasticizing nature of these compounds, which lowers intermolecular friction and weakens secondary interactions, such as van der Waals forces, between chains. Among the tested additives, lanolin exhibits the strongest plasticizing effect, resulting in the highest MFI (0.931 cm^3^/10 min). This outcome is consistent with its chemical structure, consisting of long aliphatic esters and alcohols capable of inserting between polymer chains and reducing their cohesive interactions. Lecithin and thymol show moderate increases, while cetyl alcohol exhibits a balanced effect between plasticisation and interfacial reinforcement, as reflected in its intermediate MFI value (0.674 cm^3^/10 min).

The rheological results, therefore, highlight a dual behavior: biochar increases melt stiffness and restricts flow, while natural additives, especially lanolin, counteract this effect by improving melt flow and potentially enhancing processability.

### 3.2. Viscosity of the Composites

**Figure 2:**
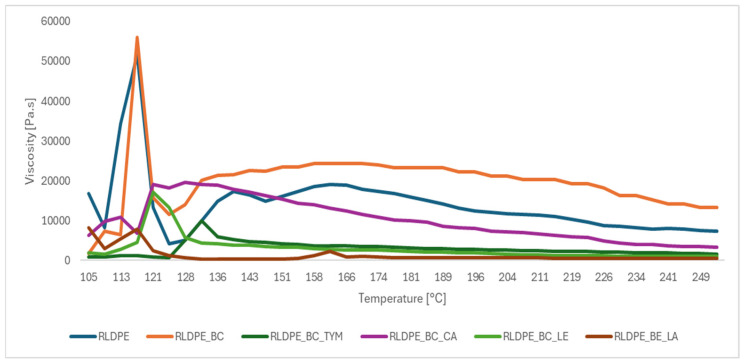
Viscosity–temperature curves of RLDPE and its composites with biochar and natural plasticizing additives.

The viscosity–temperature profiles of the analyzed composites provide further insight into the effect of biochar and natural processing-enhancing additives on the rheological behavior of regranulated LDPE and are presented in Figure 2. The reference RLDPE exhibits a typical sharp viscosity drop in the vicinity of its melting transition, followed by a gradual decrease in viscosity with increasing temperature, characteristic of thermoplastic melt thinning. The introduction of biochar (RLDPE_BC) significantly alters this behavior: the composite displays a pronounced viscosity peak near the onset of melting and maintains considerably higher viscosity values across the entire measured temperature range. This confirms that biochar increases the structural rigidity of the melt and restricts macromolecular mobility, consistent with the MFI results. Similar alterations in melt rheological behavior, including increased viscosity and modified flow characteristics due to restricted polymer chain mobility induced by biochar–polymer interactions, have been reported for biochar-filled polymer systems [37].

When natural additives are incorporated into the biochar-filled matrix, the viscosity curves shift downward, indicating a partial recovery of melt flow. The strongest reduction is observed for the lanolin-modified composite (RLDPE_BE_LA), which achieves the lowest viscosity over most of the examined temperatures. This behavior reflects the marked plasticizing action of lanolin, likely associated with its ability to penetrate between polymer chains and locally decrease cohesive interactions. Lecithin and thymol also lower viscosity relative to the biochar-only sample, although to a lesser extent, suggesting moderate chain-lubricating effects. In contrast, cetyl alcohol (RLDPE_BC_CA) displays a more complex profile, providing only partial viscosity reduction and maintaining a more stable plateau, which may be linked to its dual role in both plasticizing the polymer matrix and reinforcing interfacial interactions with the filler.

Overall, the rheological curves support a dual mechanism consistent with the MFI analysis: biochar increases melt stiffness and viscosity, whereas natural additives—particularly lanolin—counteract this effect by enhancing melt flowability and improving processing characteristics of the regranulated LDPE composites.

### 3.3. Impact Strength

**Figure 3:**
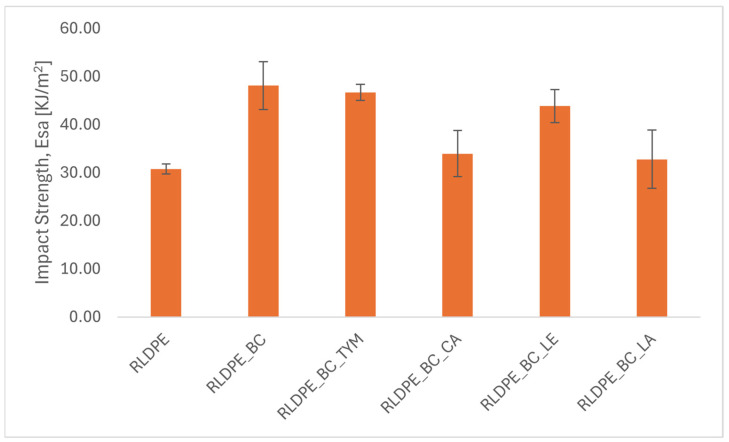
Charpy impact energy (E_sa_) of samples.

The study was conducted to determine the impact strength of materials. The measurement results are presented in Figure 3.

The impact strength tests revealed that the addition of biochar to RLDPE significantly increased impact resistance by approximately 56%, confirming the reinforcing role of biochar and the improved structural integrity of the composite. This result is consistent with the flexural and hardness data, indicating a uniform stiffening and strengthening effect.

Natural additives produced variable effects. Thymol and lecithin maintained relatively high impact strength (46.7 kJ/m^2^ and 43.8 kJ/m^2^, respectively), suggesting that these modifiers do not adversely affect impact resistance. Lanolin, however, reduced the impact strength to 32.8 kJ/m^2^, reflecting its pronounced plasticizing effect.

### 3.4. Hardness

**Figure 4:**
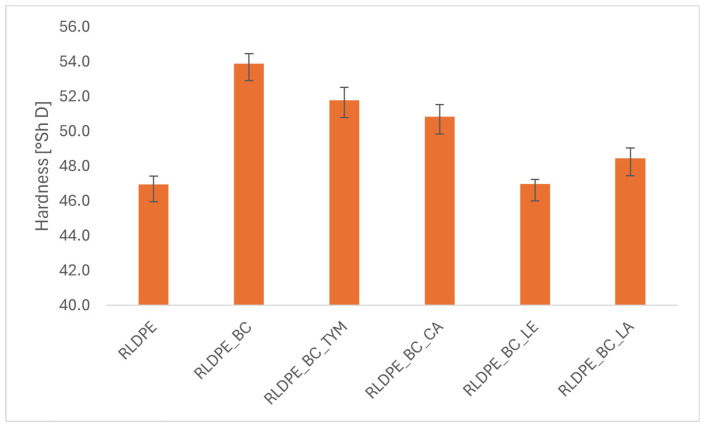
Hardness of composites.

The hardness of the obtained polyethylene composites is influenced by several factors, including the degree of dispersion of the solid phase in the polymer matrix, the level of crystallinity, and the presence of various types of modifying additives. These parameters directly affect the material’s resistance to elastic and plastic deformation, which is reflected in the hardness values measured using the Shore D method. The measurement results are presented in Figure 4.

The reference sample, consisting solely of polyethylene, exhibited the lowest hardness value (47 ± 0.5 ShD), confirming that pure polyethylene is a relatively soft material with low resistance to local surface deformation.

The addition of biochar led to an increase in hardness to 53.9 ShD, demonstrating the stiffening effect of the filler. The observed increase in hardness can be attributed to a reduction in the mobility of polymer chain segments and an increase in the proportion of the solid phase in the material.

Samples containing additional thymol and cetyl alcohol showed similarly elevated hardness values, ranging from 50.8 to 51.8 ShD. This finding indicates that the additives did not significantly affect the composite’s stiffness or plasticity.

In contrast, an alternative effect was observed in the case of lecithin, the addition of which resulted in a hardness value comparable to that of the reference sample. This may indicate that lecithin improved the dispersion of the filler in the polymer matrix, reducing the formation of agglomerates, while also exhibiting a plasticizing effect, increasing the mobility of the polymer chain segments. A comparable, albeit less significant, reduction in hardness was observed for the sample with the addition of lanolin.

### 3.5. Three-Point Bending

Table 3 summarizes the three-point bending test results, including the modulus of elasticity (Ef), maximum stress (σfM), and deformation at maximum stress (εfM).

Introducing biochar into RLDPE led to a substantial increase in stiffness, with Ef rising from 171 MPa to 303 MPa—representing an improvement of approximately 77%. The maximum stress also increased from 15.4 MPa to 18.9 MPa, confirming the pronounced reinforcing effect of biochar. The deformation at maximum stress remained nearly unchanged, indicating that the improvement in stiffness did not negatively affect the material’s elasticity. Similar improvements in mechanical performance, including increased tensile modulus (stiffness) and enhanced strength due to efficient stress transfer between the rigid biochar filler and the polymer matrix, have been reported in previous studies on biochar-filled polymer composites [19]. Among the modified composites, the sample containing cetyl alcohol exhibited the highest modulus (327 MPa) and strength (20.1 MPa), with strong interfacial reinforcement between the polymer matrix and the modified filler. However, its slightly lower elongation (12.5%) reflects a moderate reduction in ductility.

Thymol-modified samples maintained relatively high strength (18.5 MPa) but showed reduced stiffness compared with the biochar-only composite, suggesting weaker yet still effective interfacial adhesion. Lecithin and lanolin caused a noticeable decrease in both modulus and stress values, likely due to their partial plasticizing action and diminished filler–matrix interaction.

Overall, all biochar-containing composites demonstrated improved stiffness relative to pure RLDPE, with cetyl alcohol modification delivering the most significant mechanical enhancement.

### 3.6. Mechanical Properties of Composites Under Static Conditions

**Figure 5:**
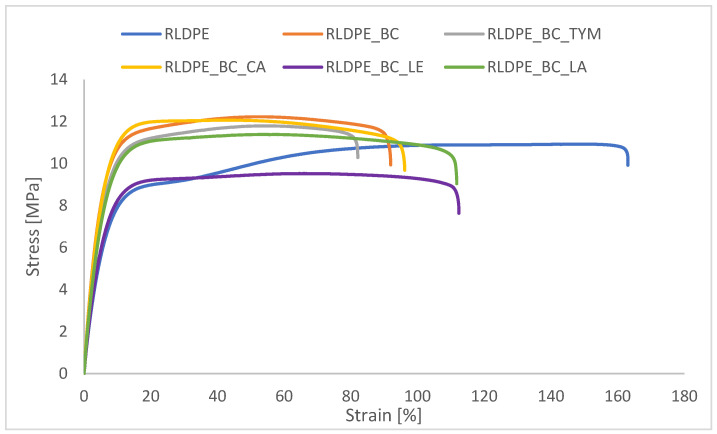
True stress and strain at the maximum load of the samples.

The true stress at maximum load (T_fmax_) and true strain at maximum load (E_fmax_) results, presented in Figure 5 and Table 4, show the influence of biochar and natural additives on the mechanical behavior of the composites.

The true stress at maximum load (Tfmax) and true strain at maximum load (Efmax) results of composites before and after aging are presented in Table 4. The addition of biochar slightly increased the true stress at maximum load (from 11.6 MPa to 12.2 MPa) but significantly reduced the true strain at maximum load (from approximately 150% to 54%), indicating a marked loss of ductility and increased brittleness resulting from restricted polymer chain mobility. Thymol and cetyl alcohol had a minimal effect on Tfmax and Efmax values before aging, producing results comparable to the biochar-filled composite. Lanolin enhanced ductility without significantly compromising strength, confirming its moderate plasticizing behavior. In contrast, lecithin caused a noticeable reduction in true stress at maximum load, suggesting limited interfacial compatibility and possible phase separation.

After thermo-oxidative aging, the mechanical response of the composites varied depending on the applied additive. Neat RLDPE exhibited a pronounced reduction in Efmax, while maintaining nearly unchanged Tfmax, indicating embrittlement caused by oxidative degradation of the polymer matrix. A similar trend was observed for the RLDPE_BC composite, reflected by a reduced aging factor (K = 0.63), confirming a deterioration of ductility after aging.

Composites modified with thymol and cetyl alcohol demonstrated improved resistance to aging, as evidenced by increased Efmax values and aging factors greater than unity (K = 1.26 and 1.22, respectively). This suggests that these additives mitigated thermo-oxidative degradation, likely by enhancing chain mobility and limiting oxidative damage. Lecithin-containing composites showed only minor changes in mechanical properties after aging (K = 1.06), indicating moderate stabilization but no significant improvement in strength. Lanolin-modified composites also exhibited enhanced elongation after aging (K = 1.13), confirming its plasticizing effect and its ability to partially counteract aging-induced embrittlement.

Overall, the aging factor analysis indicates that selected bio-based additives, particularly thymol and cetyl alcohol, can effectively improve the thermo-oxidative aging resistance of biochar-filled RLDPE composites.

### 3.7. Differential Scanning Calorimetry (DSC) Analysis

Table 5 presents the results of the differential scanning calorimetry (DSC) analysis, including the onset temperature (T_onset_), peak temperature (T_peak_), end temperature (T_endset_), enthalpy change (∆H), and degree of crystallinity (Xc).

The onset temperature of crystallization remains similar for most samples, ranging from 94 to 98 °C. A notable deviation is observed for the composite containing cetyl alcohol, where T_onset_ decreases to 88.17 °C, indicating facilitated nucleation and the initiation of crystallization at lower temperatures. The crystallization peak temperature for most samples falls between 113 and 115 °C, whereas the cetyl alcohol–modified composite exhibits a higher T_peak_ (119.45 °C), suggesting enhanced crystal growth. The endset temperatures remain within a narrow range of 124–126 °C for all samples.

Both RLDPE foil and reprocessed RLDPE exhibit comparable crystallinity values of approximately 39%, indicating that the recycling and remelting steps did not significantly affect the crystalline structure of the polymer matrix. The addition of biochar leads to a slight increase in crystallinity (39.9%), suggesting a weak heterogeneous nucleating effect of the carbonaceous filler. Such behavior has been previously reported for carbon-based fillers in polyethylene systems. A more pronounced increase in Xc is observed when biochar is combined with low-molecular-weight additives. Composites containing thymol and lecithin show crystallinity values of approximately 43.9% and 43.6%, respectively, which may be attributed to improved chain mobility and enhanced interfacial interactions facilitating crystal growth. The highest crystallinity is recorded for the composite modified with cetyl alcohol (50.4%), indicating a strong nucleating and ordering effect. This behavior can be associated with the long aliphatic chain of cetyl alcohol, which is structurally compatible with polyethylene and promotes chain alignment during crystallization. The lanolin-containing composite also exhibits elevated crystallinity (45.2%), reflecting the combined influence of ester functionalities and hydrocarbon segments on crystallization kinetics.

It should be noted that no distinct glass transition could be identified on the DSC thermograms, which is attributed to the broad and diffuse nature of the glass transition in semicrystalline polyethylene-based materials. Therefore, the DSC results primarily reflect changes in crystallization behavior rather than providing direct evidence of plasticization. For completeness, all DSC thermograms of the investigated composites are provided as Supplementary Materials.

### 3.8. Fourier-Transform Infrared Spectroscopy (FTIR) Analysis

**Figure 6:**
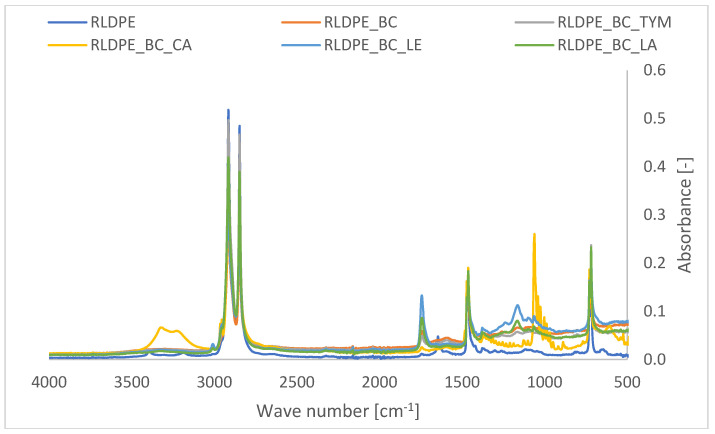
Comparative FTIR spectra of neat RLDPE and composited with biochar and processing additives.

FTIR spectra of recycled low-density polyethylene (RLDPE) and its biochar-filled composites with various processing additives were analyzed to evaluate structural changes and interfacial interactions (Figure 6). Neat RLDPE exhibits characteristic polyethylene absorption bands, including strong C–H stretching vibrations at 2915 and 2848 cm^−1^, CH_2_ bending vibrations at 1470–1460 cm^−1^, and a rocking band at 720–730 cm^−1^, confirming the preservation of the polyethylene backbone after recycling.

The incorporation of biochar does not significantly alter the characteristic bands of RLDPE, indicating the absence of chemical degradation during processing. However, a slight increase in absorbance around 1600 cm^−1^ and in the 1200–1000 cm^−1^ region is observed, corresponding to aromatic C=C and C–O stretching vibrations originating from oxygen-containing functional groups on the biochar surface [29,30,31,32,33,34,35,36,37,38,39]. These features suggest physical interactions between the polymer matrix and biochar.

The addition of lecithin results in the appearance of a carbonyl band at approximately 1730–1740 cm^−1^ and enhanced absorption in the 1050–1150 cm^−1^ region, attributed to ester and C–O–P vibrations, confirming its effective incorporation. Composites containing lanolin also exhibit an intensified carbonyl band near 1730 cm^−1^ and a broad O–H stretching band around 3300–3400 cm^−1^, indicating the presence of ester groups and weak hydrogen bonding interactions.

For cetyl alcohol-modified composites, increased intensity of the O–H stretching band (3300–3400 cm^−1^) and enhanced C–H stretching vibrations are observed, reflecting the aliphatic nature of the additive and its good compatibility with the RLDPE matrix. The presence of thymol is confirmed by additional aromatic bands in the 1510–1600 cm^−1^ region and a broad phenolic O–H stretching band, while no significant shifts in polyethylene-related bands are detected.

Overall, the FTIR results demonstrate that the chemical structure of RLDPE remains unchanged in all composites. The observed spectral features confirm the successful incorporation of biochar and processing additives, with interactions being predominantly physical in nature and contributing to improved interfacial compatibility without chemical modification of the polymer matrix.

### 3.9. Surface Energy

Table 6 presents the surface energy values for the tested samples. Surface energy is a key parameter that characterizes the interaction of a material with its environment. This includes its wettability, adhesion and potential compatibility with other substances or material layers.

The surface energy value of pure RLDPE is 28.31 mN/m, which is characteristic of hydrophobic materials with low polarity. The addition of biochar does not lead to any significant changes, indicating that biochar does not efficiently modify surface polarity.

However, it is evident that significant disparities arise when additives are used. The application of thymol resulted in an increase in surface energy to 31.10 mN/m. Conversely, an opposing trend is observed for the sample with cetyl alcohol, where the surface energy decreases to 23.12 mN/m. Cetyl alcohol, a long-chain compound, tends to migrate to the surface, thereby reducing its polarity and resulting in a notable decrease in surface energy. This phenomenon is characteristic of hydrophobic surfactants.

However, the most significant increase in surface energy is observed after the application of lecithin and lanolin, where the values are 50.95 mN/m and 45.36 mN/m, respectively. Such substantial increases indicate a strong effect of these additives on the surface properties of the composite.

Lecithin, a phospholipid with dual characteristics, exhibits a natural tendency to orient its polar phosphoric group towards the surface, thereby increasing its polarity. In comparison, lanolin, a mixture of esters and alcohols with a wide spectrum of functional groups, also contributes to an increase in surface energy, although to a slightly lesser extent than lecithin.

## 4. Conclusions

The conducted study demonstrates that both biochar and natural processing-enhancing additives substantially influence the thermal, rheological, mechanical, surface, and aging properties of regranulated LDPE. DSC analysis showed that biochar reduces the crystallinity of the composite by restricting chain mobility, whereas selected natural additives—most notably cetyl alcohol—significantly promote crystallization by facilitating nucleation and improving molecular ordering. This is reflected in the markedly increased enthalpy of crystallization and crystallinity degree observed for the cetyl-alcohol-modified composite (Xc = 50.4%).

Melt flow and rheological measurements revealed a dual behavior: biochar markedly increases melt viscosity and stiffness, while natural additives partially counteract this effect through plasticizing or lubricating interactions. Lanolin exhibited the strongest plasticizing effect, resulting in the highest melt flow and the greatest viscosity reduction, indicating improved processability of the composite.

Mechanical testing confirmed the reinforcing effect of biochar, evidenced by an increased flexural modulus and strength. Among the modified systems, the composite containing cetyl alcohol exhibited the most pronounced improvement in stiffness and overall mechanical performance, suggesting enhanced interfacial interactions as inferred from macroscopic mechanical results. However, the addition of biochar significantly reduced ductility, as indicated by the decreased elongation at break. In contrast, lanolin improved ductility without a substantial loss of strength, demonstrating its moderate plasticizing characteristics.

Hardness and impact strength measurements further supported the stiffening effect of biochar, while natural additives modulated this behavior according to their chemical nature. Thymol and cetyl alcohol maintained elevated hardness levels, whereas lecithin and lanolin induced a reduction, consistent with their partial plasticizing action. In terms of impact resistance, biochar increased energy absorption capacity, while lanolin, due to its strong softening effect, resulted in a decline.

Thermo-oxidative aging assessments revealed that thymol and cetyl alcohol effectively improved the post-aging mechanical performance of the composites, with aging factors (K) exceeding unity, while lanolin enhanced ductility after aging. Lecithin provided only minor stabilization. These results indicate that bio-based additives can mitigate aging-induced embrittlement and enhance the durability of biochar-filled RLDPE.

FTIR analysis confirmed the physical incorporation of additives and biochar, showing predominantly physical interactions and the absence of chemical degradation of the polymer matrix. Surface energy analysis revealed that biochar had a negligible influence on surface polarity, whereas natural additives produced significant changes. Lecithin and lanolin markedly increased surface energy due to their polar functional groups, enhancing surface polarity, while cetyl alcohol decreased surface energy through migration of hydrophobic aliphatic chains to the surface.

Surface energy analysis revealed that biochar had a negligible influence on surface polarity, whereas natural additives produced significant changes. Lecithin and lanolin markedly increased surface energy due to their polar functional groups, enhancing surface polarity, while cetyl alcohol decreased surface energy through migration of hydrophobic aliphatic chains to the surface.

Overall, biochar is an effective reinforcing filler for RLDPE, while natural additives enable fine-tuning of composite performance, including enhanced resistance to thermo-oxidative aging. Cetyl alcohol provides the strongest enhancement of crystallinity and mechanical strength, lanolin improves processability and ductility, and lecithin or thymol allows for controlled modification of surface and mechanical properties. Tailored combinations of biochar and selected natural additives yield RLDPE composites with improved functionality, application-specific performance, and enhanced long-term stability.

## Figures and Tables

**Table 1:** Composition of the Mixtures.

Sample	Quantity [pbw]				
	Biochar (BC)	Lecithin (LE)	Lanoline (LA)	Cetyl Alcohol (CA)	Thymol (TYM)
RLDPE	-	-	-	-	-
RLDPE_BC	20	-	-	-	-
RLDPE_BC_TYM	20	-	-	-	5
RLDPE_BC_CA	20	-	-	5	-
RLDPE_BC_LE	20	10	-	-	-
RLDPE_BC_LA	20	-	10	-	-

**Table 2:** Melt-flow rates (MVR and MFR) of composites.

Sample	MVR [cm^3^/10 min]	MFR [g/10 min]
RLDPE	0.732 ± 0.02	0.517 ± 0.02
RLDPE_BC	0.556 ± 0.08	0.419 ± 0.06
RLDPE_BC_TYM	0.762 ± 0.04	0.524 ± 0.03
RLDPE_BC_CA	0.674 ± 0.02	0.510 ± 0.01
RLDPE_BC_LE	0.766 ± 0.04	0.591 ± 0.03
RLDPE_BC_LA	0.931 ± 0.03	0.709 ± 0.02

**Table 3:** The three-point bending test results, including the modulus of elasticity (Ef), maximum stress (σfM), and deformation at maximum stress (εfM).

Sample	Ef [MPa]	σfM [MPa]	εfM [%]
RLDPE	171 ± 27.9	15.4 ± 1.8	13.5 ± 1.0
RLDPE_BC	303 ± 18.5	18.9 ± 1.8	13.6 ± 0.2
RLDPE_BC_TYM	277 ± 10.5	18.5 ± 1.3	13.1 ± 0.2
RLDPE_BC_CA	327 ± 31.9	20.1 ± 0.3	12.5 ± 0.4
RLDPE_BC_LE	258 ± 15.8	15.9 ± 0.6	13.6 ± 0.1
RLDPE_BC_LA	249 ± 10.9	16.9 ± 0.3	13.6 ± 0.5

**Table 4:** True stress (Tfmax) and true strain (Efmax) at maximum load of the composites before and after thermo-oxidative aging, including the aging factor (K).

Sample	Before Aging		After Aging		K
	T_fmax_ [MPa]	E_fmax_ [%]	T_fmax_ [MPa]	E_fmax_ [%]	
RLDPE	11.6 ± 0.6	149.6 ± 31.0	11.6 ± 0.5	101.2 ± 6.3	0.68
RLDPE_BC	12.2 ± 0.5	54.1 ± 4.7	12.0 ± 0.4	34.4 ± 5.2	0.63
RLDPE_BC_TYM	11.8 ± 0.1	54.8 ± 4.5	12.7 ± 0.6	64.2 ± 3.7	1.26
RLDPE_BC_CA	11.9 ± 0.2	45.5 ± 5.4	11.5 ± 0.1	57.3 ± 4.1	1.22
RLDPE_BC_LE	9.6 ± 0.1	65.8 ± 3.6	10.0 ± 0.1	67.3 ± 2.3	1.06
RLDPE_BC_LA	11.1 ± 0.2	55.7 ± 2.4	10.8 ± 0.2	64.7 ± 4.7	1.13

**Table 5:** The thermal characteristics of composites were determined by DSC analysis.

Sample	T_onset_ [°C]	T_peak_ [°C]	T_endset_ [°C]	∆H [J/g]	Xc [%]
RLDPE_Foil	94.52	113.84	125.60	114.8	39.2
RLDPE	94.06	114.17	126.22	114.4	39.0
RLDPE_BC	98.66	115.98	125.98	97.4	39.9
RLDPE_BC_TYM	95.95	114.05	125.15	102.8	43.9
RLDPE_BC_CA	88.17	119.45	124.44	118.1	50.4
RLDPE_BC_LE	97.28	115.51	125.56	98.3	43.6
RLDPE_BC_LA	97.54	114.73	124.60	101.9	45.2

**Table 6:** Surface energy for composites.

Sample	Surface Energy [mN/m]
RLDPE	28.31
RLDPE_BC	28.25
RLDPE_BC_TYM	31.10
RLDPE_BC_CA	23.12
RLDPE_BC_LE	50.95
RLDPE_BC_LA	45.36

## Data Availability

The original contributions presented in this study are included in the article/Supplementary Material. Further inquiries can be directed to the corresponding author.

## Supplementary Materials

The following supporting information can be downloaded at https://www.mdpi.com/article/10.3390/ma19030570/s1, Figure S1: DSC thermograms of the RLDPE sample recorded during the heating and cooling cycles. Figure S2: DSC thermograms of the RLDPE/biochar composite recorded during the heating and cooling cycles. Figure S3: DSC thermograms of the RLDPE/biochar/lanolin composite recorded during the heating and cooling cycles. Figure S4: DSC thermograms of the RLDPE/biochar/lecithin composite recorded during the heating and cooling cycles. Figure S5: DSC thermograms of the RLDPE/biochar/cetyl alcohol composite recorded during the heating and cooling cycles. Figure S6: DSC thermograms of the RLDPE/biochar/thymol composite recorded during the heating and cooling cycles.

## Abbreviations

The following abbreviations are used in this manuscript:

RLDPE	Regranulated low-density polyethylene
BC	Biochar
LE	Lecithin
LA	Lanoline
CA	Cetyl Alcohol
TYM	Thymol
MFI	Melt Flow Index
MFR	Melt Flow Rate
MVR	Melt Volume Rate
PN-EN ISO	Polish Standard—European Standard—International Organization for Standardization
DSC	Differential Scanning Calorimetry
T_fmax_	True Stress at Maximum Load
E_fmax_	True Strain at Maximum Load
Xc	Crystalline Phase Content
K	Aging Factor
Ef	Elasticity
σfM	Maximum Stress
εfM	Deformation at Maximum Stress
T_onset_	Onset Temperature
T_peak_	Peak Temperature
T_endset_	End Temperature
∆H	Enthalpy Change
