# Supplementary figures and images for: The Use of Bioadditives as Plasticizers in Recycled Polyethylene Materials
Source: Materials (Basel). 2026 Feb 2;19(3):570. doi: 10.3390/ma19030570 (PMC12897606; doi:10.3390/ma19030570)

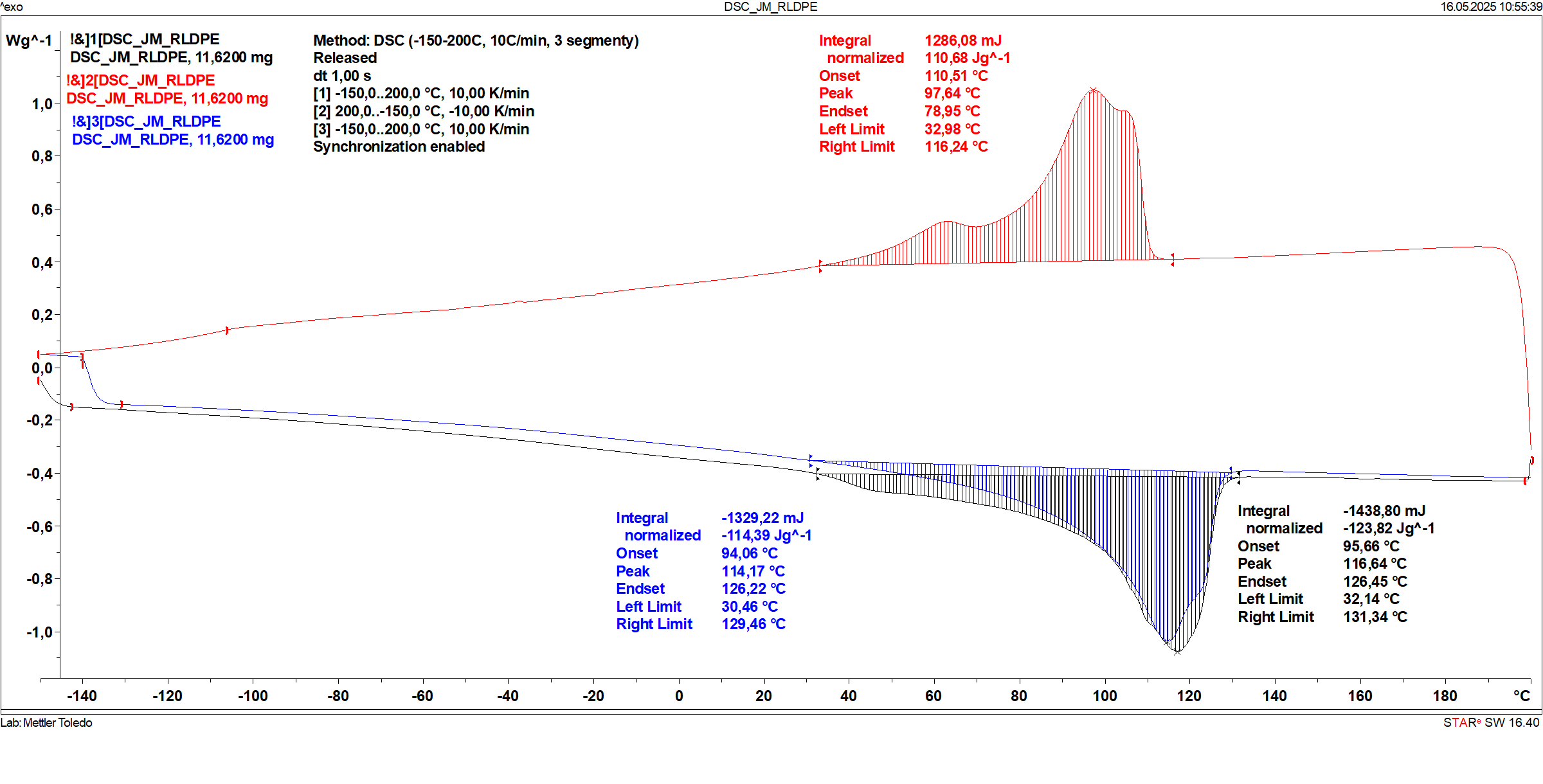

Supplement: Supplementary file 1 [file materials-19-00570-s001.zip › Figure S1-DSC_JM_RLDPE.tif]

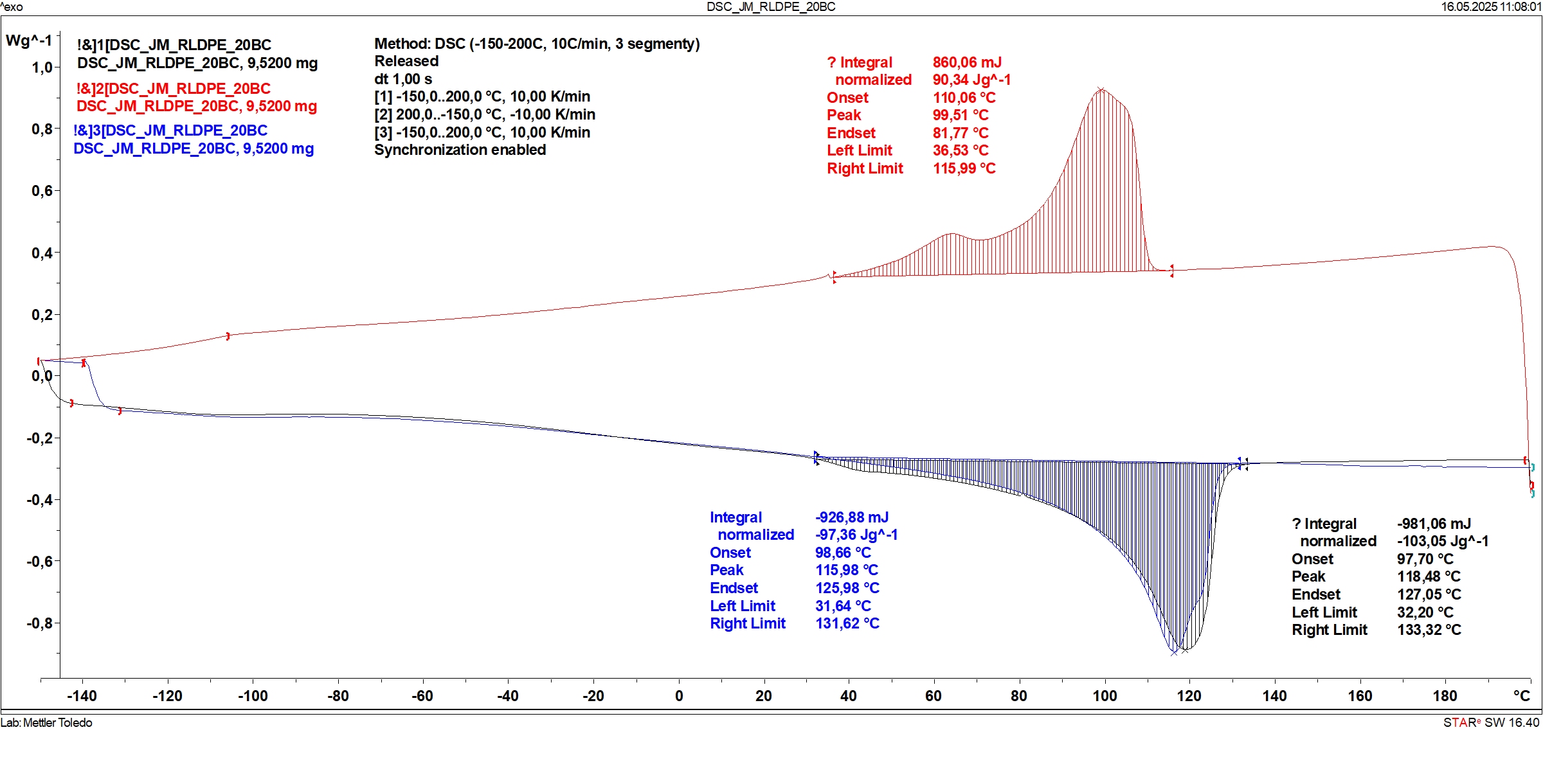

Supplement: Supplementary file 1 [file materials-19-00570-s001.zip › Figure S2-DSC_JM_RLDPE_20BC.tif]

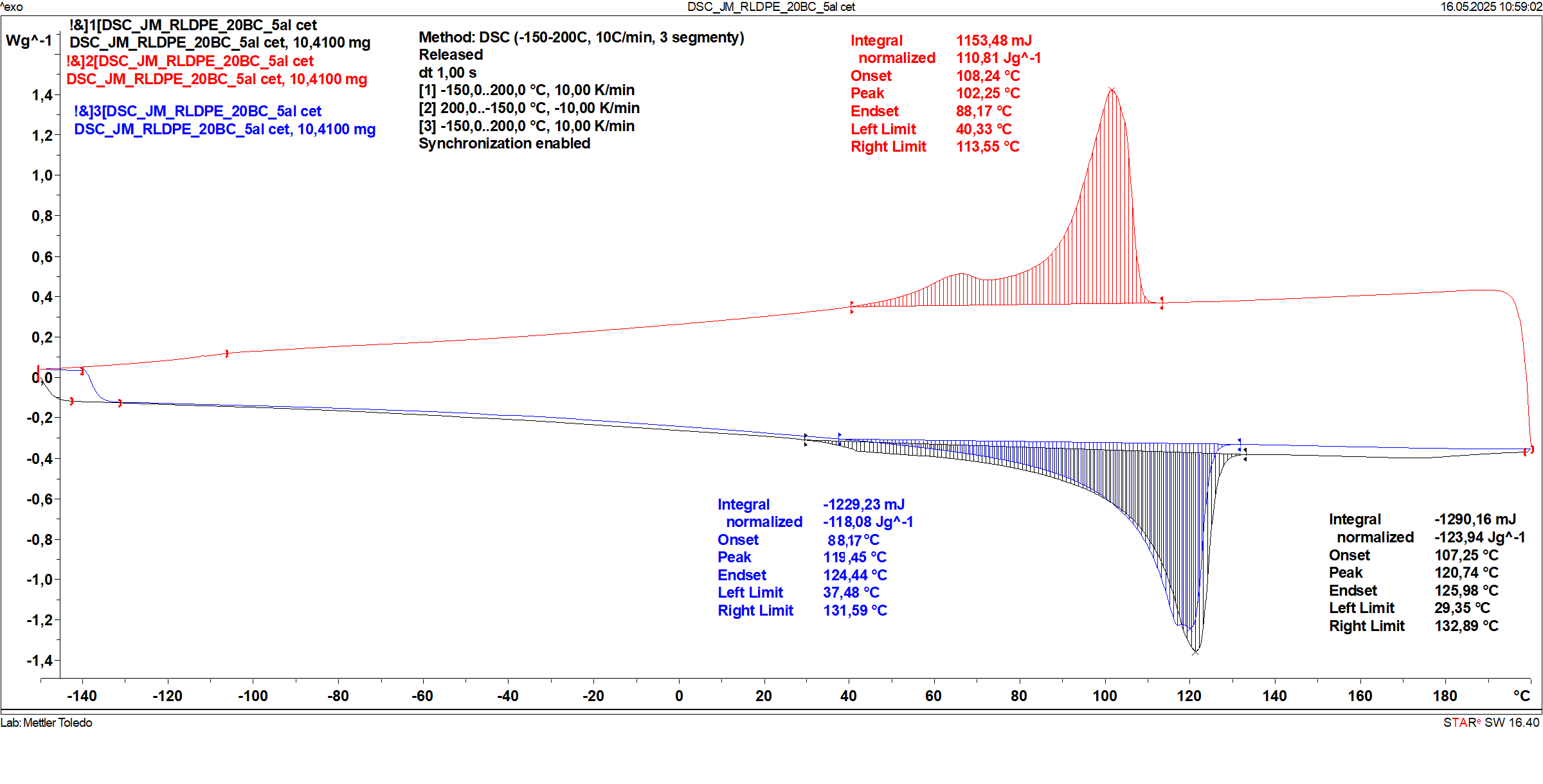

Supplement: Supplementary file 1 [file materials-19-00570-s001.zip › Figure S3-DSC_JM_RLDPE_20BC_5al cet.tif]

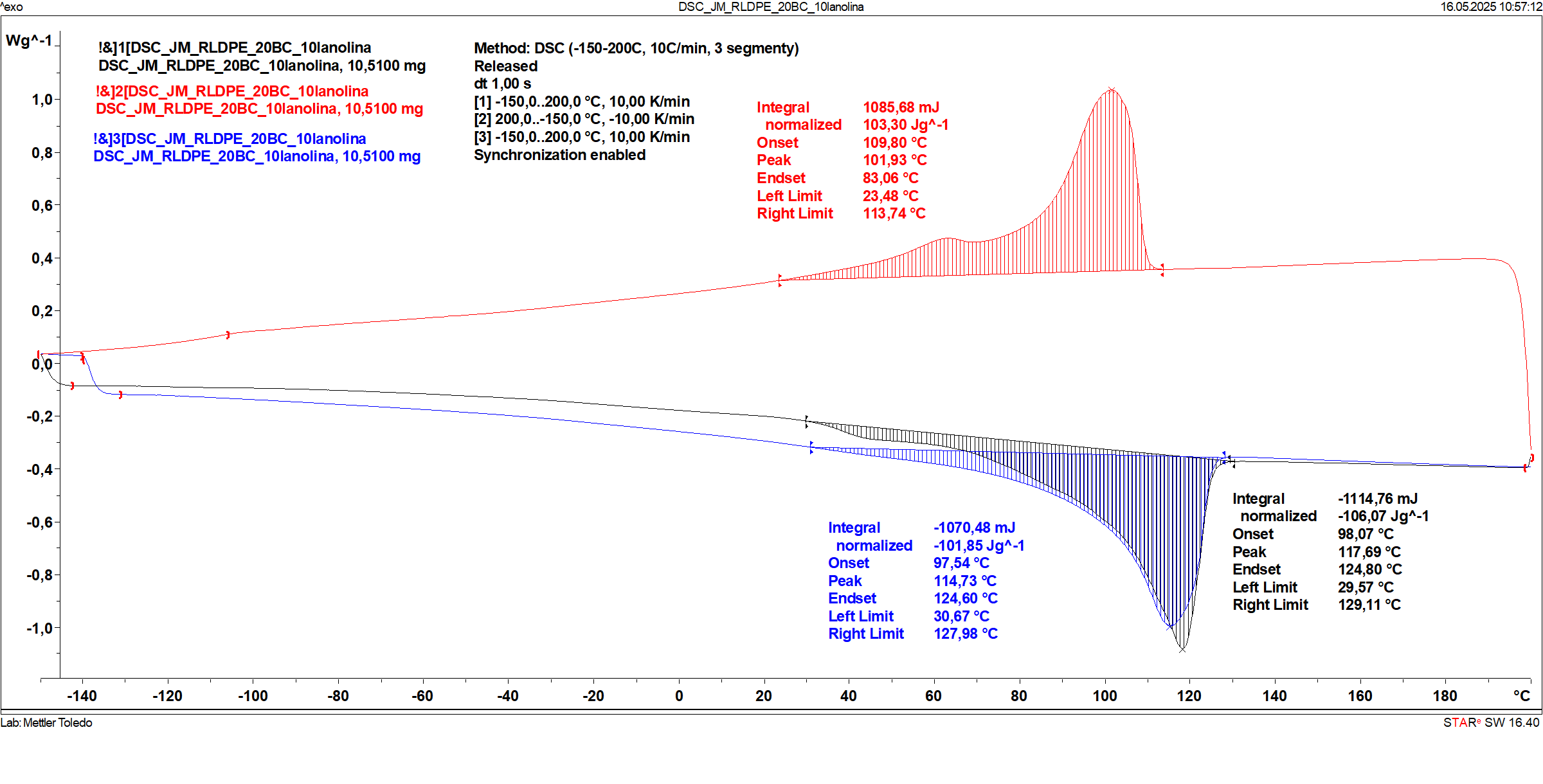

Supplement: Supplementary file 1 [file materials-19-00570-s001.zip › Figure S4-DSC_JM_RLDPE_20BC_10lanolina.tif]

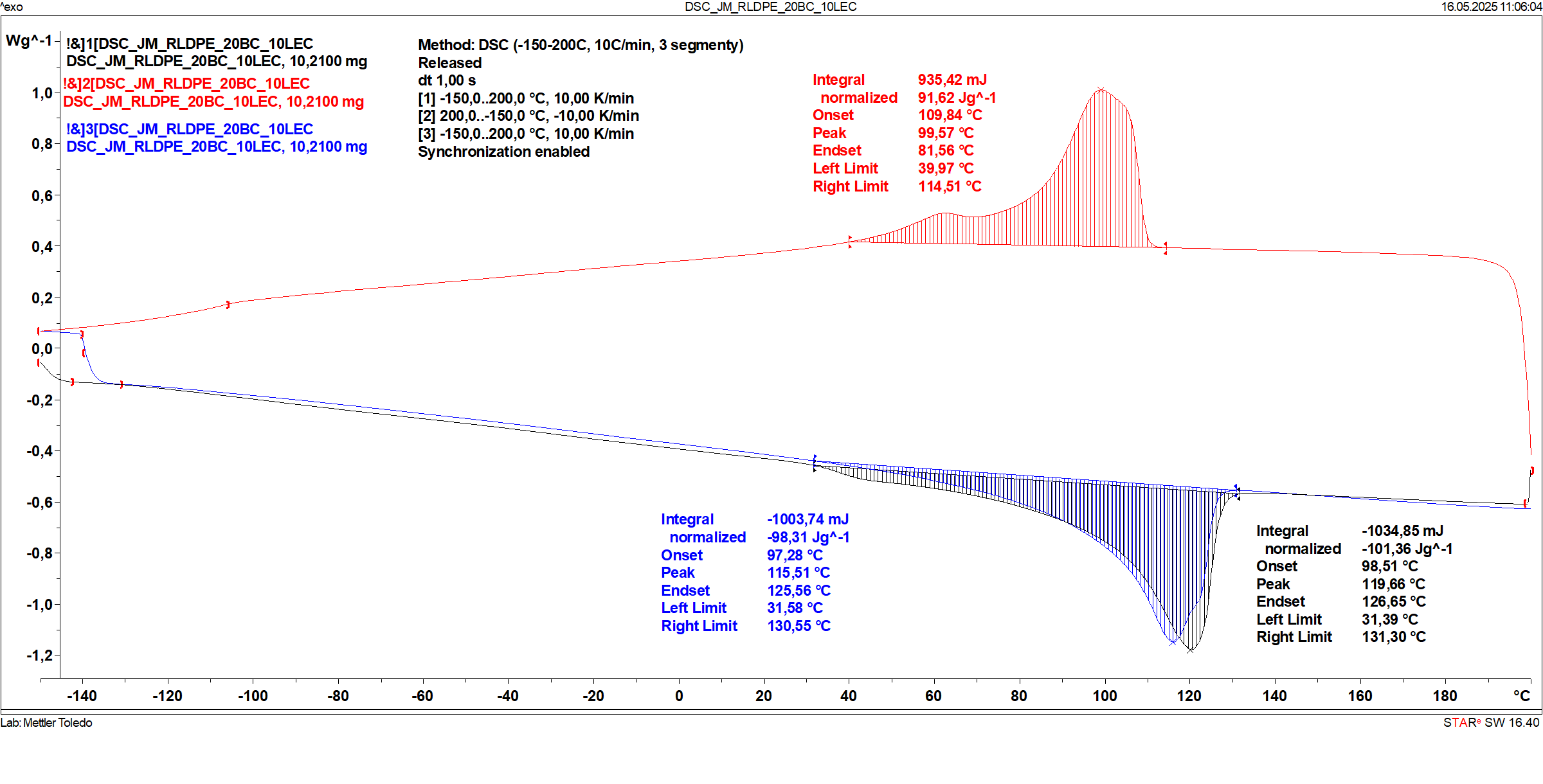

Supplement: Supplementary file 1 [file materials-19-00570-s001.zip › Figure S5-DSC_JM_RLDPE_20BC_10LEC.tif]

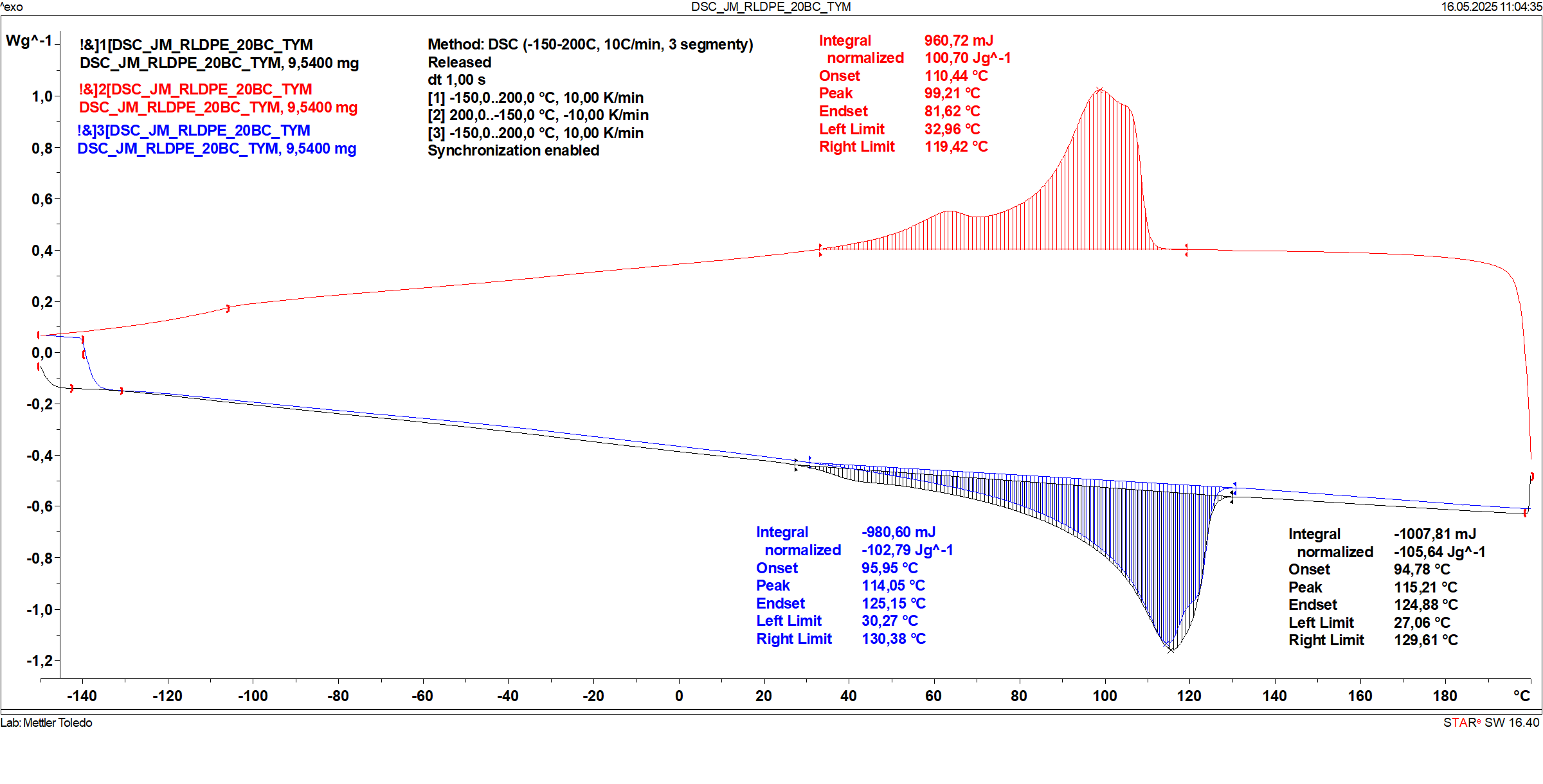

Supplement: Supplementary file 1 [file materials-19-00570-s001.zip › Figure S6-DSC_JM_RLDPE_20BC_TYM.tif]
